# Terrestrial invasion of pomatiopsid gastropods in the heavy-snow region of the Japanese Archipelago

**DOI:** 10.1186/1471-2148-11-118

**Published:** 2011-05-05

**Authors:** Yuichi Kameda, Makoto Kato

**Affiliations:** 1 Graduate School of Human and Environmental Studies, Kyoto University, Yoshida-nihonmatsu-cho, Sakyo, Kyoto 606-8501, Japan

## Abstract

**Background:**

Gastropod mollusks are one of the most successful animals that have diversified in the fully terrestrial habitat. They have evolved terrestrial taxa in more than nine lineages, most of which originated during the Paleozoic or Mesozoic. The rissooidean gastropod family Pomatiopsidae is one of the few groups that have evolved fully terrestrial taxa during the late Cenozoic. The pomatiopsine diversity is particularly high in the Japanese Archipelago and the terrestrial taxa occur only in this region. In this study, we conducted thorough samplings of Japanese pomatiopsid species and performed molecular phylogenetic analyses to explore the patterns of diversification and terrestrial invasion.

**Results:**

Molecular phylogenetic analyses revealed that Japanese Pomatiopsinae derived from multiple colonization of the Eurasian Continent and that subsequent habitat shifts from aquatic to terrestrial life occurred at least twice within two Japanese endemic lineages. Each lineage comprises amphibious and terrestrial species, both of which are confined to the mountains in heavy-snow regions facing the Japan Sea. The estimated divergence time suggested that diversification of these terrestrial lineages started in the Late Miocene, when active orogenesis of the Japanese landmass and establishment of snowy conditions began.

**Conclusions:**

The terrestrial invasion of Japanese Pomatiopsinae occurred at least twice beside the mountain streamlets of heavy-snow regions, which is considered the first case of this event in the area. Because snow coverage maintains stable temperatures and high humidity on the ground surface, heavy-snow conditions may have paved the way for these organisms from freshwater to land via mountain streamlets by preventing winter desiccation in mountain valleys. The fact that the terrestrialization of Pomatiopsidae occurred only in year-round wet environments, but not in seasonally dried regions, provides new insight into ancient molluscan terrestrialization.

## Background

Terrestrial invasion is one of the most important events in the history of life [1,2]. Terrestrial lineages evolved in seven animal phyla, among which vertebrates, arthropods, and mollusks are representatives of more successful groups of the epifaunal terrestrial life [3]. Most terrestrial lineages are considered to have originated under mild or tropical climate during the Paleozoic and Mesozoic, whereas few animals became fully terrestrial during the Cenozoic [3,4]. In regard to this point, mollusks are especially unique due to several fully terrestrial and epifaunal lineages that evolved during the Cenozoic [5,6].

Terrestrial invasion of gastropod mollusks has occurred in Neritopsina, Cyclophoroidea, Littorinoidea, Rissooidea, Ellobioidea, Onchidioidea, Rathouisioidea, Succineoidea, and Stylommatophora [7,8], and in particular, each of Neritopsina, Rissooidea and Ellobioidea has likely achieved land invasion more than once [6,8,9]. Most terrestrialization events have occurred during the Paleozoic or Mesozoic [2,8-10], and only some members of rissooidean families Truncatellidae, Assimineidae, and Pomatiopsidae are considered to have colonized to land during the Cenozoic [5,6,11,12]. Most truncatellid and assimineid snails amphibiously live in intertidal and supratidal zones from brackish water to pelagic areas. Terrestrial lineages likely evolved from such ancestors [4,6]. In contrast, most pomatiopsid snails live in freshwater habitats [11,13]. Therefore, the process of terrestrial invasion in Pomatiopsidae might differ from those of former families.

**Figure 1:**
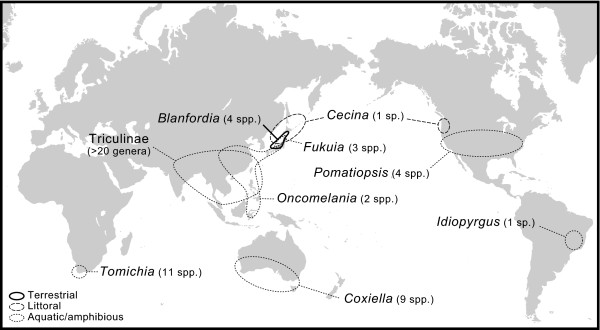
**A map showing worldwide distribution of Pomatiopsine genera and Triculinae (after Davis **[11]**)**. Terrestrial taxa occur only on the Japanese Archipelago located in East Asia (*Blanfordia*). *Tomichia *and *Coxiella *include several halophilic species occuring on saline lakes.

**Figure 2:**
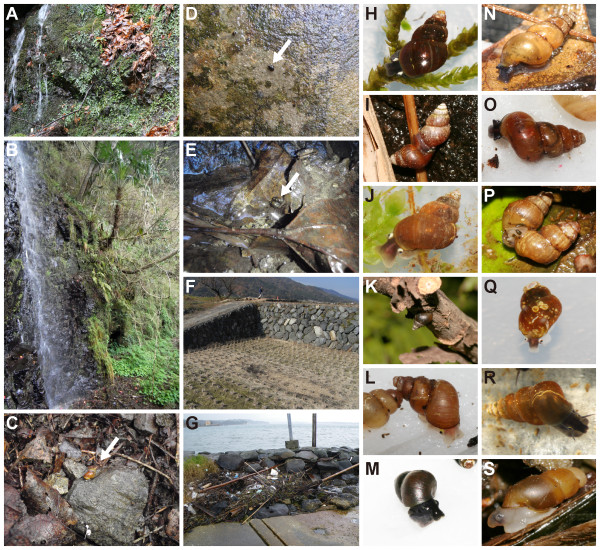
**Japanese pomatiopsine snails and their habitats**. **A**. Dripping rocks beside the waterfall covered with bryophytes, inhabited by amphibious species *F. kurodai*. **B**. Habitats of amphibious species *F. multistriata *(beside the waterfall) and terrestrial species *B. simplex *(forest floor). **C**. Terrestrial species *B. japonica *on the forest floor. **D**. Aquatic species *F. ooyagii *clinging to wet rocky wall. **E**. Aquatic species *O. minima *inhabiting mountain streamlet. **F**. Rice field (dry season) inhabited by seasonally amphibious species *O. h. nosophora*. **G**. Decaying seaweed stranded on the beach, inhabited by *C. manchurica*. **H**. *F. kurodai niigataensis*. **I**. *F. k. kurodai *mating outside the water film. **J**. *F. multistriata*. **K**. *F. multistriata *resting on a tree branch. **L**. *B. integra *(terrestrial). **M**. *F. ooyagii*. **N**. *B. *sp. (terrestrial). **O**. *B. bensoni *(terrestrial). **P**. *B. simplex *mating on the forest floor. **Q**. *O. minima*. **R**. *O. h. nosophora*. **S**. *C. manchurica *found among decaying seaweeds.

Pomatiopsidae, which are well known as intermediate hosts of Asian schistosomes [14-18], comprises two subfamilies, Triculinae and Pomatiopsinae. The former radiated as aquatic snails in freshwater habitats in Southeast Asia [11,19], whereas the latter are distributed worldwide (Figure 1) and possesses various lifestyles from aquatic to amphibious, littoral, halophilic, and even terrestrial [11,20]. The generic diversity of Pomatiopsinae is particularly high in the Japanese Archipelago, where four of the eight genera including two endemics, are recorded (Figures 1 &2) [11]. The diversity of Japanese Pomatiopsinae is based on two characteristics, i.e. unique habitats of the endemic genera, and their distribution.

**Figure 3:**
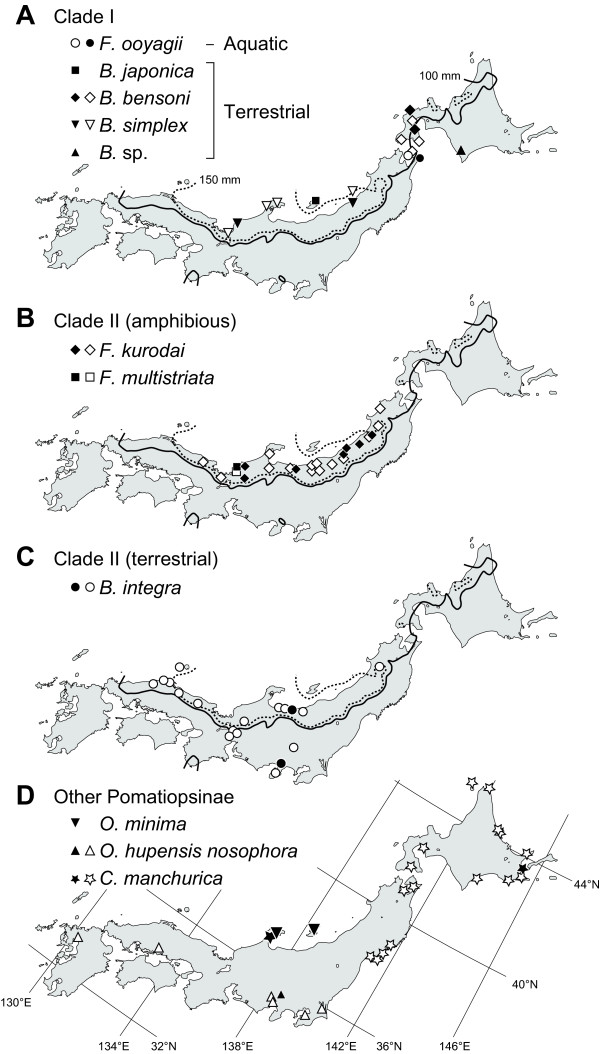
**Maps of Japan showing distributions of Pomatiopsine taxa**. (a) Clade 1; (b) amphibious species of clade 2, *Fukuia kurodai *and *F. multistriata*; (c) terrestrial species of clade 2, *B. integra*; (d) other pomatiopsine species. Solid and open symbols represent the collecting sites in this study and distribution record from the literature, respectively [22,35,72-76]. Solid and dashed curves indicate areas that have more than 100 mm and 150 mm, respectively, of total precipitation in January (average of 1971-2000 [77]).

One of the two Japanese endemic genera, *Fukuia*, live amphibiously around rocky walls of steep valleys covered with ferns and bryophytes, and moistened by dripping water [21,22]. They inhabit only along the mountain streamlets, where such habitats are typically found, and often occur with pleurocerid freshwater snails [21]. In contrast, the other endemic genus *Blanfordia *is the sole terrestrial group within Pomatiopsidae (Figure 1) [11]. They are found on forest floor and tree trunks in coastal to mountainous areas (Table 1) [11,22], together with fully terrestrial gastropod snails such as diplommatinids, camaenids and bradybaenids. The distribution of the two genera are largely confined to the heavy-snow region facing the Japan Sea (Figures 3A-C) [22]. Generally, East Asia is dominated by a monsoon climate, characterized by alternating hot rainy summers and cold dry winters [23]. The snow-rich area is unique in that winter monsoons blowing southward from the Japan Sea and high mountain ranges shaping the Japanese Archipelago bring enormous snowfalls in winter [24,25]. Such a varied climate harbors various unique flora and fauna [26,27], and can be closely related to the diversification of Pomatiopsinae in the archipelago.

**Table 1:** Sampling information of specimens used in the present study

Taxon	Habitat	Locality	18S	28S	16S	COI
POMATIOPSIDAE						
Pomatiopsinae						
*Blanfordia bensoni *(Adams, 1861)	Inland forests; terrestrial	Nigorikawa, Mori, Hokkaido	AB611708*	AB611709*	AB611710*	AB611711*
		Futoro, Setana, Hokkaido	AB611712*	AB611713*	AB611714*	AB611715*
*Blanfordia integra *Pilsbry, 1924	Inland forests; terrestrial, often arboreal	Mt. Mikuni, Shizuoka	AB611716*	AB611717*	AB611718*	AB611719*
		Nou, Itoigawa, Niigata	AB611720*	AB611721*	AB611722*	AB611723*
*Blanfordia japonica *(Adams, 1861)	Coastal to inland forests; terrestrial	Sado, Niigata^1^	AB611724*	AB611725*	AB611726*	AB611727*
*Blanfordia simplex *Pilsbry, 1902	Coastal dunes and littoral forests; terrestrial	Yunohama, Tsuruoka, Yamagata^1^	AB611728*	AB611729*	AB611730*	AB611731*
		Ayukawa, Fukui, Fukui	AB611732*	AB611733*	AB611734*	AB611735*
*Blanfordia *sp.	Inland forests; terrestrial	Samani, Hokkaido	AB611736*	AB611737*	AB611738*	AB611739*
*Cecina manchurica *Adams, 1861	Littoral	Nanao, Ishikawa	AB611740*	AB611741*	AB611742*	AB611743*
		Betsukai, Hokkaido	AB611744*	AB611745*	AB611746*	AB611747*
*Fukuia kurodai kurodai *Abbott & Hunter, 1949	Mountain streamlets; amphibious, often arboreal	Ohyana, Yurihonjo, Akita	AB611748*	AB611749*	AB611750*	AB611751*
		Atsumi, Tsuruoka, Yamagata	AB611752*	AB611753*	AB611754*	AB611755*
		Kitamata, Sakata, Yamagata	AB611756*	AB611757*	AB611758*	AB611759*
		Shimouchinami, Ohno, Fukui	AB611760*	AB611761*	AB611762*	AB611763*
		Takeda, Maruoka, Sakai, Fukui^1^	AB611764*	AB611765*	AB611766*	AB611767*
*Fukuia kurodai niigataensis *Minato, 1973	Mountain streamlets; amphibious, often arboreal	Oyachi, Itoigawa, Niigata	AB611768*	AB611769*	AB611770*	AB611771*
		Ikazuchi, Murakami, Niigata	AB611772*	AB611773*	AB611774*	AB611775*
*Fukuia multistriata *Abbott & Hunter, 1949	Mountain streamlets; amphibious, often arboreal	Umeura, Echizen, Fukui	AB611776*	AB611777*	AB611778*	AB611779*
*Fukuia ooyagii *Minato, 1982	Mountain streamlets; aquatic	Iwaya, Mutsu, Aomori^1^	AB611780*	AB611781*	AB611782*	AB611783*
*Oncomelania hupensis nosophora *(Robson, 1915)	Lakes/marshy ground; seasonally amphibious	Nirasaki, Yamanashi	AB611784*	AB611785*	AB611786*	AB611787*
*Oncomelania hupensis quadrasi *(Möllendorff, 1895)	Lakes/marshy ground; seasonally amphibious	Genbank	-	-	DQ212862	DQ112287
*Oncomelania hupensis robertsoni *Bartsch, 1946	Lakes/marshy ground; seasonally amphibious	Genbank	AF212906	AY207042	AF212893	AF213339
*Oncomelania minima *(Bartsch, 1936)	Mountain streamlets; aquatic	Wajima, Ishikawa	AB611788*	AB611789*	AB611790*	AB611791*
		Sado, Niigata	AB611792*	AB611793*	AB611794*	AB611795*
*Pomatiopsis lapidaria *(Say, 1817)	Marshy ground; amphibious	Genbank	AF367666	-	AY676118	AF367636
Triculinae						
*Gammatricula fujianensis *(Liu, Zhang & Wang, 1983)	Aquatic	Genbank	AF212909	-	AF212896	AF213342
*Gammatricula shini *(Habe, 1961)	Aquatic	Yonaguni Isl., Okinawa	AB611796*	AB611797*	AB611798*	AB611799*
*Gammatricula songi *Davis, Chen & Yu, 1994	Aquatic	Genbank	-	EF394879	EF394867	EF394902
*Lacunopsis *sp.	Aquatic	Genbank	AF212910	-	AF212897	AF213343
*Neotricula aperta *(Temcharoen, 1971)	Aquatic	Genbank	AF531540	AY207034	AF531556	AF531541
*Neotricula burchi *(Davis, 1968)	Aquatic	Genbank	AF531543	AY207035	AF531542	AF531544
*Robertsiella *sp.	Aquatic	Genbank	AF531549	-	AF531548	AF531550
*Tricula bollingi *Davis, 1968	Aquatic	Genbank	AF531552	AY207039	AF531551	AF531553
*Tricula fujianensis *(Liu, et al., 1983)	Aquatic	Genbank	-	EF394885	EF394873	EF394893
*Tricula hongshanensis *Tang et al., 1986	Aquatic	Genbank	-	EF394888	EF394876	EF394896
*Tricula hortensis *Attwood et al., 2003	Aquatic	Genbank	-	EF394883	EF394871	EF394900
*Tricula hsiangi *Kang, 1984	Aquatic	Genbank	-	EF394889	EF394877	EF394897
*Tricula pingi *Kang, 1984	Aquatic	Genbank	-	EF394881	EF394869	EF394901
*Tricula wumingensis *Hu et al., 1994	Aquatic	Genbank	-	EF394884	EF394872	EF394892
ASSIMINEIDAE						
*Angustassiminea satumana *Habe, 1942		Kikai Isl., Kagoshima	AB611800*	AB611801*	AB611802*	AB611803*
*Assiminea hiradoensis *Habe, 1942		Urakami, Nagasaki, Nagasaki	AB611804*	AB611805*	AB611806*	AB611807*
*Paludinellassiminea japonica *(Pilsbry, 1901)		Hiburi Isl., Uwajima, Ehime	AB611808*	AB611809*	AB611810*	AB611811*
*Pseudomphala miyazakii *(Habe, 1943)		Ashikari, Ogi, Saga	AB611812*	AB611813*	AB611814*	AB611815*
TRUNCATELLIDAE						
*Truncatella pfeifferi *Martens, 1860		Nanao, Ishikawa	AB611816*	AB611817*	AB611818*	AB611819*
AMNICOLIDAE						
*Akiyoshia kobayashii *Kuroda & Habe, 1958		Taga, Shiga	AB611820*	AB611821*	AB611822*	AB611823*
LITTORINIDAE						
*Echininus cumingi spinulosus *(Philippi, 1846)		Kikai Isl., Kagoshima	AB611824*	AB611825*	AB611826*	AB611827*
*Littorina pallescens *(Philippi, 1846)		Nago, Okinawa	AB611828*	AB611829*	AB611830*	AB611831*

^1^Type locality

Taxon names, habitats, localities, and accession numbers are provided. Sequences obtained for this study are marked with an asterisk.

In this study, we provide the first molecular phylogeny encompassing all pomatiopsid taxa occurring on the Japanese Archipelago. We also analyzed the relationship with continental taxa. In addition, we estimated the timing of diversification and terrestrial invasion, which can be interpreted using the geological history of Japan. Based on these results, we discuss the reason why the terrestrial invasion of Pomatiopsidae occurred only in the Japanese Archipelago.

## Results

### Phylogenetic analyses

To investigate the evolutionary history of pomatiopsid snails in the Japanese Archipelago, we sequenced the mitochondrial cytochrome oxidase subunit 1 (CO1), 16S ribosomal RNA genes, and nuclear 18S and 28S rRNA regions (Table 1). Maximum parsimony (MP) analysis of the combined data resulted in a single most parsimonious tree (3375 steps, consistency index excluding uninformative characters [CI] = 0.3861, retention index [RI] = 0.6567). The results of maximum likelihood (ML) and Bayesian analyses were largely consistent with the relatively well supported clades. Only posterior probabilities of *P *≥ 0.95 and bootstrap support values of *P *≥ 75 were considered statistically significant. Hence, support values below this significance level are not discussed.

**Figure 4:**
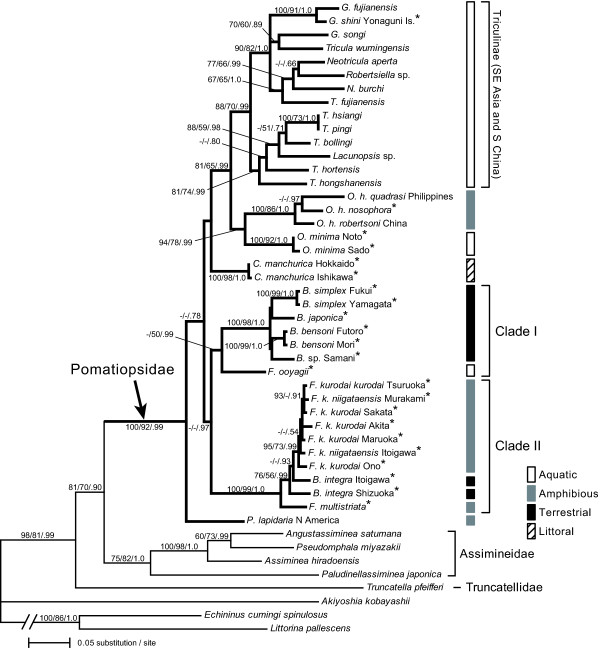
**A maximum likelihood tree based on the combined dataset of 18S, 28S, 16S, and CO1 genes (-ln likelihood = 20667.0486)**. Numbers on branches indicate maximum parsimony and maximum likelihood bootstrap values followed by Bayesian posterior probabilities. Bars on the right of the tree indicate habitat of each taxon. Japanese pomatiopsid taxa are indicated by asterisks. Clades I and II are endemic to Japanese Archipelago.

The phylogenetic analyses recovered Pomatiopsidae as a monophyletic clade (Figure 4). Triculinae was also confirmed as monophyletic. On the other hand, relationships among most of the pomatiopsine genera are not well resolved, although the pomatiopsine genus *Oncomelania *was sister to Triculinae. Following analysis using an approximately unbiased(AU) test [28], the monophyly of all pomatiopsine taxa was rejected (*P *= 0.049), suggesting that Pomatiopsinae is paraphyletic to Triculinae.

Japanese pomatiopsine taxa were grouped into four lineages. *Oncomelania *and *Cecina *were both recovered as monophyletic, but two endemic genera, *Fukuia *and *Blanfordia *formed two clades, each of which included species from both genera (clades I and II; Figure 4). Clade I consisted of *F. ooyagii *and *Blanfordia *spp. except for *B. integra*, where *F. ooyagii *was sister to the *Blanfordia *species. Clade II was composed of *B. integra*, *F. kurodai*, and *F. multistriata*. These results indicate independent evolution of terrestriality in the two clades. Therefore, we tested the alternative hypothesis of the occurrence of terrestrial species. The monophyly of all *Blanfordia *species was rejected by approximately unbiased (AU) test (*P *= 0.000001), suggesting that land invasion occurred independently at least twice. In clade II, *B. integra *was placed between *F. multistriata *and *F. kurodai*. However, the detailed branching pattern and number of terrestrial invasions were uncertain within this clade because the monophyly of *B. integra *was not rejected (AU test, *P *= 0.07).

### Estimation of divergence time

**Figure 5:**
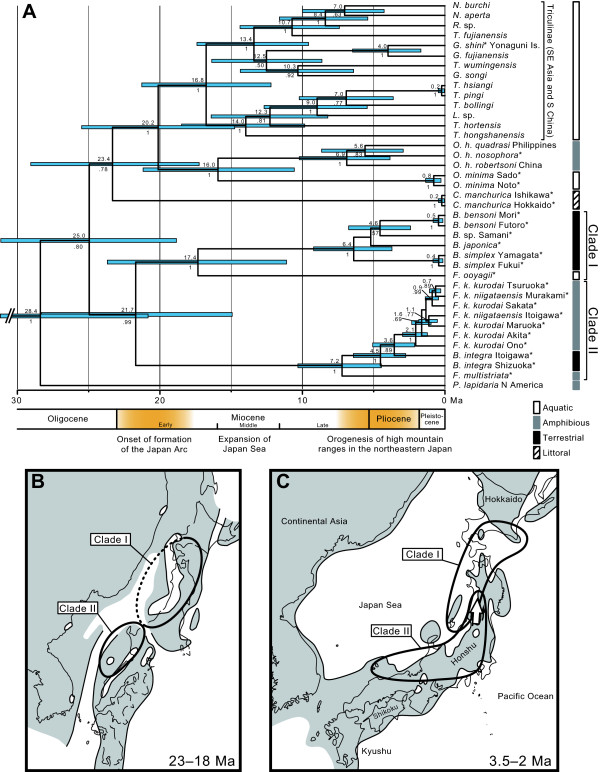
**Maximum clade probability tree displayed as a chronogram and paleogeography around the Japanese Archipelago**. (A) Divergence times calculated in BEAST based on fossil records (outgroups not shown). Japanese pomatiopsid taxa are indicated by asterisks. Horizontal bars in the tree give the 95% credible interval for the age of each node. Numbers above and below branches represent mean node ages and posterior probabilities, respectively. (B, C) Hypothesized paleodistribution of clades I and II. Paleogeographic maps of East Asia were modified from Iijima & Tada [37]. Shaded areas indicate land. (B) Early Miocene (23-18 Ma), divergence of two clades. (C) Middle to late Pliocene (3.5-2 Ma), ongoing diversification and range expansion within each clade.

To determine the timing of the origin of terrestriality in Pomatiopsidae, we estimated the divergence time based on the combined phylogeny of four genes. Fossil records and previous estimations were used to estimate the divergence time of pomatiopsid taxa. For the fossil records, *Assiminea *spp. from the Lower Miocene (23.0-16.0 Ma) [29,30] were used for the initial divergence of Assimineidae. The root node of "Truncatelloidea" (Truncatellidae + Pomatiopsidae + Assimineidae; sensu Ponder [31]) was modeled by a Gamma prior (shape = 1 and scale = 1.5) with values younger than 61.7 Ma assigned zero probability, which is justified by *Truncatella minor*, the oldest known fossil for this group documented from the Lower Paleocene (65.5-61.7 Ma) of Belgium [32]. Based on these constraints, a basal split within Triculinae was inferred at 16.8 Ma with a 95% confidence interval of 21.3-12.2 Ma (Figure 5A). The emergence of Japanese aquatic species *O. minima *and *F. ooyagii *were estimated at 16.0 (21.2-10.6) Ma and 17.4 (23.7-11.1) Ma, respectively. The terrestrial lineage in clade I began to diverge at 6.4 (9.2-3.7) Ma. The diversification of clade II began at 7.2 (10.3-4.4) Ma and the terrestrial species *B. integra *occurred during the Pliocene.

We also estimated the divergence time by using the split between *Oncomelania hupensis quadrasi *and *O. h. robertsoni*, which was previously estimated at 3 Ma based on allozyme differences [33]. Node ages calculated from this estimation were two to three times younger than former results. Thus, data from the two calibrations were not compatible at higher nodes (data not shown). Although we could not determine which estimation is more accurate, we employed the former results because the node ages in the latter estimation were too young to explain the existence of fossils at earlier periods.

### Climatic factors contributing to distribution of endemic genera

The two Japanese endemic lineages, clades I and II, are largely confined to the Japan Sea side of the Japanese Archipelago (Figure 3). To determine whether this pattern is associated with any climate factors, we performed logistic regression analysis based on four variables, i.e., annual highest and lowest temperatures, and total precipitations in summer and winter. The logistic regression analysis showed significant correlation between winter precipitation and the occurrence of the snails, whereas contributions of the other variables were not significant (Table 2). Although we did not include the amount of snowfall due to the significant correlation with winter precipitation (Spearman's rank correlation coefficient, *p *= 0.0054), its difference was also significant between inside and outside of the distributions (Wilcoxon's signed-rank test, *z *= -3.009, *p *= 0.0026).

**Table 2:** Chi-square statistics from the logistic regression analysis

Variables	χ^2^	P
Highest temperature	1.6651 × 10^-9^	1
Lowest temperature	9.7577 × 10^-7^	0.9992
Summer precipitation	2.9000 × 10^-8^	0.9999
Winter precipitation	34.7674	< 0.0001

Degrees of freedom was 1 for all variables.

## Discussion

### Phylogenetic and taxonomic implications

The phylogenetic analyses supported the monophyly of Triculinae. On the other hand, Pomatiopsinae was paraphyletic to the former subfamily, and an Asian genus *Oncomelania *became sister to Triculinae (Figure 4). These results suggest that taxonomy and phylogeographic scenarios should be reconsidered. Traditionally, Pomatiopsidae is considered to have diverged into two subfamilies in Gondwanaland during the Mesozoic, mainly because three pomatiopsine genera shows Gondwanan distribution (*Coxiella*, *Idiopyrgus *and *Tomichia*; Figure 1) [11]. However, the close relationship between Triculinae and *Oncomelania *and the estimated divergence time suggest that Triculinae have evolved more recently on the Eurasian Continent, not in Gondwanaland. Although we could not provide alternative scenarios from current phylogenetic results, further study, including remaining pomatiopsine genera, is needed to reconsider subfamilial taxonomy and worldwide phylogeography of this family.

Incongruence between current taxonomy and molecular phylogeny was also observed on Japanese endemic genera *Fukuia *and *Blanfordia*. The terrestrial species *B. integra *was grouped with the amphibious species *F. kurodai *and *F. multistriata*, whereas aquatic species *F. ooyagii *was separated from the other *Fukuia *species (Figure 4). Our results indicated the inadequacy of the current taxonomy of the two genera, which have been primarily based on their habitat [34]. In contrast, internal morphologies likely reflect the phylogenetic relationship. Davis [11] noted that genital structures of *B. integra *are similar to those of *Fukuia*. Although he doubted if *Fukuia *and *Blanfordia *are morphologically distinct based on such evidence, it is an apparent confusion resulting from the inadequate generic assignment of *B. integra*, for genital and nervous structures clearly differ between species in clade I and II [11,21]. Snails in clade II also commonly climb up grasses and shrubs during summer (Figure 2K) [11, 35, Y. Kameda, pers. obs.]. This behavior characterizes clade II, since tree-climbing behavior is known only in these snails and a few arboreal species of the assimineid genus *Omphalotropis *throughout the superfamily [36]. Hence, *B. integra *is undoubtedly a member of the genus *Fukuia*. At the same time, *F. ooyagii *should be separated from *Fukuia*, and its generic assignment should be determined coupled with the investigation of its soft-part morphology.

### Divergence time and patterns of Japanese Pomatiopsinae

The number of inhabiting pomatiopsine genera is particularly high in the Japanese Archipelago (Figure 1). Davis [11] considered that the diversity of Japanese Pomatiopsinae results from the single colonization and following radiation of ancestral *Oncomelania *in the archipelago, and that such rapid evolution presumably associated with extensive tectonic activity in the Tertiary. However, our phylogenetic analysis grouped Japanese pomatiopsine snails into four lineages, *Oncomelania*, *Cecina*, and clades I and II (Figure 4). These results contradict the scenario of Davis [11], suggesting that current diversity is partially derived from the multiple colonization of pomatiopsine ancestors into the Japanese Archipelago. Subsequent habitat shifts may have contributed to increasing diversity and produced the terrestrial lineages.

The widespread genus *Oncomelania *comprises two Japanese taxa, *O. minima *and *O. h. nosophora *(Figures 2Q &2R). These two taxa are not closely related, suggesting that colonization to the Japanese Archipelago occurred twice in this genus. The age of divergence between *O. minima *and *O. hupensis *was estimated around 16.0 Ma in the Middle Miocene (Figure 5A), suggesting that this divergence was likely invoked by the expansion of the Japan Sea, which is thought to have occurred during this period [37,38]. *O. h. nosophora *diverged from other continental subspecies more recently and likely colonized into Japan during glacial periods in the Late Pleistocene [39].

The littoral genus *Cecina *also occurs in the Japanese Archipelago. This genus consists of a single widespread species, *Cecina manchurica*, living among the decaying seaweed stranded on the seashore (Figures 2G &2S) [11]. Although we could not elucidate the divergence time of this genus due to low support values, the wide distribution was formed by recent dispersions via ocean currents based on very low genetic diversity between geographically distant populations (Figures 3D &4).

The divergence between two Japanese endemic lineages, clades I and II, was estimated to be around 21.7 Ma in the Early Miocene (Figure 5A). This period corresponds to incipient opening of the Japan Sea, and the northern and southern parts of the Japanese Archipelago that were separated from each other [37,38,40]. This geological event might have also split the two clades (Figure 5B). The northern part of the Japanese Archipelago was further fragmented at later periods until the orogenesis of the northeastern Japanese Archipelago began in the later Late Miocene [37,41]. Speciation in clade I may be explained by geographic isolation due to this geological event, and the narrow distribution of each species may reflect the past geographic isolation (Figures 3A &5C). Remarkably, *B. simplex *is widely distributed along the Japan Sea and occurs on coastal dunes as well as littoral forests, whereas the other species inhabit rather inland forests [22,42]. The low genetic differentiation and habitat of *B. simplex *suggest that a geological barrier has been absent for the coastal species.

The southern part of the Japanese Archipelago, where clade II was thought to have inhabited (Figure 5B), remained as a relatively large landmass. The land area had expanded northward in the Late Miocene along with the beginning of orogenesis in the northeastern part of the archipelago [37,38]. The diversification of clade II likely started in the Late Miocene (around 7.2 Ma; Figure 5A), indicating that tectonic activities might have enabled the range expansion of clade II to northeastern Honshu (Figure 5C).

### Routes of terrestrial invasion

**Figure 6:**
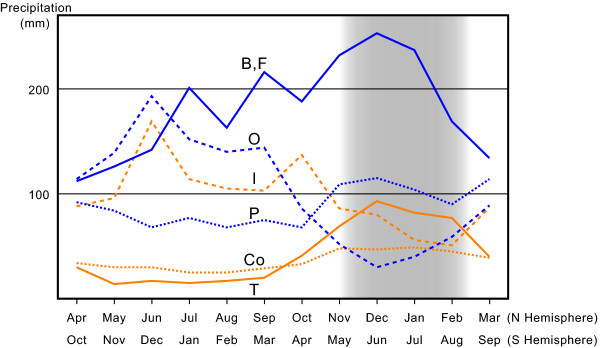
**Monthly precipitations in the distributions of pomatiopsine genera**. Values for each genus, except for *Idiopyrgus *and *Tomichia*, are average of 5 localities which was randomly selected from the distribution. The precipitation data were obtained from the World Meteorological Organization (WMO; http://www.wmo.int/). Blue and orange lines represent genera in Northern and Southern Hemisphere, respectively. Distribution of *Fukuia *and *Blanfordia *has especially high precipitation during winter (shaded period). B, *Blanfordia*; Co, *Coxiella*; F, *Fukuia*; I, *Idiopyrgus*; O, *Oncomelania*; P, *Pomatiopsis*; T, *Tomichia*.

The terrestrial pomatiopsid species are found in clades I and II (Figure 4). The snails in both clades are largely restricted to mountains in the Japan Sea side of the Japanese Archipelago (Table 1; Figures 3A-C), where winter precipitations are especially high among the distributions of pomatiopsine genera (Figure 6). Because winter precipitation falls as snow in this region, terrestrial invasion may have occurred via mountain streamlets that are covered with heavy snowfall in winter. This evolutionary hypothesis contrasts with the current knowledge of terrestrial invasion in two points. First, evolution of terrestriality most likely occurred via mountain streamlets. Generally, that animals would evolve terrestriality near rivers and streams than in lakes and swamps seems less likely due to adaptations for air breathing, particularly in the latter environment with its frequent reductions in dissolved oxygen [3]. Second, terrestrial invasion likely occurred in a cool-temperate region. Colonization to land is considered to have occurred primarily in the tropics during mild climate periods [3,4]. To our knowledge, this is the first case in which terrestriality might have evolved in a cool region. Therefore, the questions on how terrestriality was acquired beside the streamlets and which climatic factors enabled this evolution under such cold climates remain to be elucidated.

The aquatic and amphibious species of clades I and II, which likely represent ancestral habitats of terrestrial taxa, inhabit mountain streamlets and rocky walls that are constantly moistened by dripping water and covered with ferns and bryophytes (Figure 2) [21,22]. When these habitats become drier, both amphibious and aquatic species are able to resist the short-term drying by crawling into crevices and wet litter [21]. Correlation between desiccation tolerance and wetness of habitat is observed in some animals [11,43,44]; thus, frequent droughts may have promoted the ancestral freshwater snails to the more amphibious lifestyle beside the mountain streamlets. Amphibious snails in some populations can actively move onto terrestrial substrata and mate outside the water (Figure 2I). Therefore, the colonization to terrestrial habitats was presumably achieved by becoming more tolerant to desiccation. The availability of dissolved oxygen might not have influenced the habitat shifts of these snails because their ancestors may have preadapted to amphibious habitats [11,12].

The snails in clades I and II mainly occur on the Japan Sea side of the Japanese Archipelago (Figure 3), whose climate is unique in East Asia. This area is generally dominated by a monsoon climate, characterized by alternating hot rainy summers and cold dry winters [23]. However, the Japan Sea side of the archipelago has exceptionally high winter precipitation due to the winter monsoon blowing southward from the Japan Sea and the high mountain ranges that bring enormous snowfall [24,25,45]. This region harbors various unique flora and fauna that are known as "Japan Sea elements" [26,27], whose distributions clearly correspond to the amount of snowfall [46-48]. These organisms are not always resistant to the stress of winter coldness and desiccation, and often need snow coverage under which stable temperatures around 0°C and high humidity are maintained [49-54]. Snails of clades I and II are also considered as Japan Sea elements [22,35]. The logistic regression analysis showed significant correlation between winter precipitation and occurrence of the snails (Table 2), suggesting a high degree of need for high winter precipitation, which brings snow coverage, to ensure their survival.

Heavy-snow environments in the Japanese Archipelago were established by the formation of Japan Sea and high mountain ranges in the archipelago [55,56]. The orogenesis of the northern Honshu occurred since the late Miocene [37,38,41], whose period corresponds to the diversification and terrestrial invasion of Japanese endemic clades (Figure 5A). According to the vegetation in the late Miocene to Pliocene, northern Honshu was cool-temperate and later became colder [57,58]. The amount of snowfall increased with the growth of high mountain ranges during the Pleistocene [56]. Although the amount of snowfall during these periods is uncertain, arid or semi-arid climates did not appear in this region [24,56], suggesting the presence of constantly moist environments that were suitable for the survival of snails in clades I and II. Therefore, the establishment of snowy regions may have paved the way for these organisms from freshwater to land via mountain streamlets by preventing winter desiccation in mountain valleys.

The fact that terrestrial invasion in Pomatiopsidae did not occur except in the snowy region provides an indication of molluscan terrestrialization. Many pomatiopsid snails lived in the floodplains of continental Asia [11], where the dry and wet seasonal cycle is dominant (Figure 6). However, such climate seems insufficient to lead the seasonal amphibious snails to terrestrial habits. In contrast, year-round wet environments were most likely necessary for amphibious snails to become terrestrial, contrary to the belief that seasonal drought accelerates terrestrial invasion [3]. The terrestrial lineages, especially in clade II, have specific behaviors when crawling up vegetation [11,35]. Ancestral amphibious lineages in water films found on wet rocky walls would have enabled the preadaptation of this type of behavior accompanied by a broad, strong foot.

## Conclusions

The generic and ecological diversity of Pomatiopsinae is particularly high in the Japanese Archipelago, with its terrestrial taxa occurring only in this region. Our phylogenetic analyses suggested that the Japanese Pomatiopsinae derived from the multiple colonization from the Eurasian Continent and subsequent habitat shift to the terrestrial life occurred at least twice within the Japanese endemic lineages (clades I and II; Figure 4). The evolution of terrestriality possibly occurred beside mountain streamlets of snowy regions, where snow coverage shelters the ground surface from winter desiccation and coldness [51,54]. The estimated divergence time also suggested that diversification of the endemic clades began in the Late Miocene, which is the period corresponding to the orogenesis of the Japanese Archipelago and expansion of the Japan Sea (Figure 5). The emergence of the heavy-snow region is associated with these tectonic activities [56]; therefore, the terrestrial invasion of pomatiopsid snails may have been promoted by the formation of the archipelago and the establishment of snowy conditions that led the way from freshwater to land via mountain streamlets. The fact that terrestrial invasions of Pomatiopsidae occurred only in year-round wet and cool environments, but not in seasonally dried regions, gives new insight into molluscan terrestrialization.

## Methods

### Sampling

To investigate the evolutionary history of Japanese pomatiopsids, we collected 13 taxa from 23 localities to represent all Japanese pomatiopsids. The sequence data of non-Japanese pomatiopsids were obtained from GenBank, but species that lack two or more examined gene regions were excluded from the phylogenetic analysis. As a result, 13 triculine species and 3 non-Japanese pomatiopsine taxa were included in the analysis, although they do not encompass representatives of all pomatiopsine genera. However, it would not matter in discussing the evolution within Japanese taxa because excluded pomatiopsine genera are unlikely related to our focusing taxa based on genital structures [11]. For outgroups, we used four assimineids, one truncatellid, one amnicolid, and two littorinids. Detailed information on examined samples is shown in Table 1.

### Molecular methods

Total DNA was isolated following a previously described method [59]. We sequenced the partial region of the nuclear 18S rDNA and 28S rDNA, mitochondrial cytochrome oxidase subunit 1 (CO1), and 16S rDNA. Polymerase chain reaction (PCR) conditions and primers used are shown in Additional File 1. PCR products were purified using Exo-SAP-IT (Amersham Biosciences, Little Chalfont, Buckinghamshire, UK). Sequencing was performed using a BigDye™ Terminator Cycle Sequencing Ready Reaction Kit (Applied Biosystems, Foster City, CA) and electrophoresed on an ABI 3130 sequencer (Applied Biosystems). The obtained sequences were deposited in the DNA Data Bank of Japan (DDBJ) (Table 1).

### Phylogenetic analyses

The alignment of the CO1 gene required no gaps; 16S, 18S, and 28S sequences were aligned using MUSCLE v3.8 [60]. To eliminate the uncertainty of the alignments in these gene regions, GBLOCKS v0.91b [61] was used to select those regions of the aligned sequences that are confidently aligned for analysis (Additional File 2). Phylogenetic trees were obtained by maximum parsimony (MP), maximum likelihood (ML), and Bayesian methods. MP analysis was performed using PAUP* 4.0b10 [62] and heuristic searches were conducted with 10 random addition analyses using equal character weights and tree-bisection-reconnection (TBR) branch swapping. Prior to ML and Bayesian analyses, we used the program Kakusan3 v3.5.2009.11.03 [63] to select appropriate models of sequence evolution (Additional File 2). Based on the selected models, ML analysis was performed with 1000 iterations of the likelihood ratchet [64,65] using TreeFinder [66] and Phylogears version 1.5.2009.12.29 [67]. Nodal support for the MP and ML analyses was assessed using bootstrap analyses with 1000 replications. Bayesian analysis was performed using MrBayes v3.1.2 [68,69]. The analysis was carried out by two simultaneous runs, each of which consisted of running four simultaneous chains for 10 million generations and sampling trees every 1000 generations. We discarded the first 1001 trees as burn-in and the remaining 18000 samples were used to estimate the tree topology, branch length, and substitution parameters. The alignments and obtained trees were deposited in TreeBASE http://www.treebase.org/treebase-web/home.html under accession number S11213.

### Approximately unbiased (AU) test

Given that the phylogenetic analyses indicated that the terrestrial genus *Blanfordia *and aquatic/amphibious genus *Fukuia *formed mixed clades (see Results), we performed the AU tests [28] to test an alternative hypothesis for the evolution of terrestrial species. The alternative trees were obtained by the likelihood ratchet method as described above under the topological constraint that all *Blanfordia *species were monophyletic (i.e., terrestrial invasion occurred once) and that *B. integra *was monophyletic, indicating that terrestriality evolved at least twice. In addition, the AU test was also performed to test the monophyly of Pomatiopsinae, because it was not recovered as monophyletic and because support values among pomatiopsine genera were low. The AU test was conducted based on 1 million replications using TreeFinder.

### Estimation of divergence time

Approximate divergence times were estimated using a relaxed clock method [70] as implemented in BEAST 1.5.3 [71]. Divergence times were calculated using previous estimations and fossil records. We first used the split between *Oncomelania hupensis quadrasi *and *O. h. robertsoni*, which was previously estimated at 3 Ma based on allozyme differences [33]. For the fossil records, three fossils were used to place priors on node ages. A normally distributed estimate prior of 19.5 ± 3.5 Ma was used for the basal divergence of Assimineidae, based on *Assiminea *from the Lower Miocene (23.0-16.0 Ma) of Florida, USA [29] and the Marshall Islands [30]. The basal splitting of "Truncatelloidea" (Truncatellidae + Assimineidae + Pomatiopsidae; sensu Ponder [31]) was modeled by a Gamma prior (shape = 1 and scale = 1.5) with values younger than 61.7 Ma assigned zero probability, which is justified by *Truncatella minor*, the oldest known fossil for this group documented from the Lower Paleocene (65.5-61.7 Ma) of Belgium [32]. Divergence times for the remaining nodes in the phylogenetic tree were estimated with BEAST software. The Yule process was used to model the speciation process. The Monte Carlo Markov chain was run eight times for 10 million generations sampled every 1000 generations to ensure that the effective sample size (ESS) values were above 200 for most of the parameters. The first 1001 trees of each run were discarded as burn-in and the remaining trees were combined to produce an ultrametric consensus tree using LogCombiner and TreeAnnotator v1.5.3 (included in the BEAST software package).

### Climatic factors contributing to distribution of endemic genera

The two Japanese endemic genera, *Fukuia *and *Blanfordia*, are largely confined to the Japan Sea side of the Japanese Archipelago, i.e., heavy-snow regions (Figure 3). The distributions of these genera seem to be restricted by the amount of snowfall, but other climatic factors may have affected the survival of these snails. Therefore we performed logistic regression analysis to investigate the association between climatic factors and the occurrence of these snails. We used annual highest and lowest temperatures, and total precipitations in summer (between June and August) and winter (between December and February) as climatic factors. The climatic data were obtained from the Japan Meteorological Agency http://www.jma.go.jp. Within the distribution of *Fukuia *and *Blanfordia*, 14 localities were selected to cover the entire range of these genera (Additional file 3). For the localities outside the distributions, we selected 11 weather stations, each of which is nearest to an inside-distribution locality across the high mountain ranges and has similar altitude.

## Authors' contributions

YK designed the study, collected material, carried out gene sequencing, performed phylogenetic analyses, and drafted the manuscript. MK conceived of the study, participated in its design, collected material and helped to draft the manuscript. All authors have read and approved the final manuscript.

## Supplementary Material

Additional file 1 **Information on primers and PCR conditions used in this study**. Click here for file

Additional file 2 **Information on sequence alignments and models of sequence evolution for maximum likelihood analysis**. Click here for file

Additional file 3 **Information on localities used for the investigation of climatic factors**. Click here for file

## References

[B1] Shear WA The early development of terrestrial ecosystems Nature 1991 351 283 289 10.1038/351283a0

[B2] Vermeij GJ Dudley R Why are there so few evolutionary transitions between aquatic and terrestrial ecosystems? Biol J Linn Soc 2000 70 541 554 10.1111/j.1095-8312.2000.tb00216.x

[B3] Little C The terrestrial invasion. An ecophysiological approach to the origin of land animals 1990 Cambridge: Cambridge University Press

[B4] Little C The colonisation of land: Origins and adaptations of terrestrial animals 1983 Cambridge: Cambridge University Press

[B5] Wenz W Pompeckj JF Gastropoda extramarina tertiaria. Nos. 4, 6 Fossilium Catalogus 1: Animalia pt. 23 1923 Berlin: Junk 1069 1420 1735-1862

[B6] Rosenberg G Independent evolution of terrestriality in Atlantic truncatellid gastropods Evolution 1996 50 682 693 10.2307/2410841 28568923

[B7] Zilch A Gastropoda, pt. 2, Euthyneura Berlin: Borntraeger 1959-1960

[B8] Baker GM Baker GM Gastropods on land: phylogeny, diversity, and adaptive morphology The Biology of Terrestrial Molluscs 2001 New Zealand: CABI Publishing 1 146

[B9] Kano Y Chiba S Kase T Major adaptive radiation in neritopsine gastropods estimated from 28S rRNA sequences and fossil records Proc R Soc Lond B 2002 269 2457 2465 10.1098/rspb.2002.2178 PMC1691182 12495489

[B10] Solem A Gray J, Boucot AJ Biogeographic significance of land snails, Paleozoic to Recent Historical Biogeography, plate tectonics, and the changing environment 1979 Oregon: Oregon State University Press 277 287

[B11] Davis GM The origin and evolution of the gastropod family Pomatiopsidae, with emphasis on the Mekong river Triculinae Monogr Acad Nat Sci Phila 1979 20 1 120

[B12] Davis GM Historical and ecological factors in the evolution, adaptive radiation, and biogeography of freshwater mollusks Am Zool 1982 22 375 395

[B13] Dillon RT Jr The Ecology of Freshwater Molluscs 2000 Cambridge: Cambridge University Press

[B14] Bartsch P Molluscan intermediate hosts of the Asiatic blood fluke, *Schistosoma japonicum*, and species confused with them Smithson Misc Collect 1936 95 1 60

[B15] Davis GM Snail hosts of Asian *Schistosoma *infecting man: evolution and coevolution Malacol Rev Suppl 1980 2 195 238

[B16] Rollinson D Southgate VR Rollinson D, Simpson AJG The genus *Schistosoma*: a taxonomic appraisal The biology of schistosomes: from genes to latrines 1987 London: Academic Press 1 49

[B17] Attwood SW Scistosomiasis in the Mekong region: epidemiology and phylogeography Adv Parasitol 2001 50 87 152 11757333 10.1016/s0065-308x(01)50030-5

[B18] Blair D Davis GM Wu B Evolutionary relationships between trematodes and snails emphasizing schistosomes and paragonimids Parasitology 2001 123 S229 S243 11769286 10.1017/s003118200100837x

[B19] Attwood SW Ambu S Meng XH Upatham ES Xu FS Southgate VR The phylogenetics of triculine snails (Rissooidea: Pomatiopsidae) from South-east Asia and southern China: Historical biogeography and the transmission of human schistosomiasis J Molluscan Stud 2003 69 263 271 10.1093/mollus/69.3.263

[B20] Williams WD Mellor MW Ecology of *Coxiella *(Mollusca, Gastropoda, Prosobranchia), a snail endemic to Australian salt lakes Palaeogeogr Palaeoclimatol Palaeoecol 1991 84 339 355 10.1016/0031-0182(91)90053-T

[B21] Abbott RT Hunter GW III Studies on potential snail hosts of *Schistosoma japonicum* Proc Helminthol Soc 1949 16 73 89

[B22] Minato H *Blanfordia japonica *group (Gastropoda: Pomatiopsidae) considered as the distributional pattern of the so-called "Japan Sea element" confined to the Japan Sea region Nanki Seibutu 1987 29 21 24

[B23] Webster PJ Magana VO Palmer TN Shukla J Tomas RA Yanai M Yasunari T Monsoons: processes, predictability, and the prospects for prediction, in the TOGA decade J Geophys Res 1998 103 14451 14510 10.1029/97JC02719

[B24] Suzuki H The classification ofJapanese climates Tirigaku Hyouron 1962 35 205 211

[B25] Ninomiya K Heat and water budget over the Japan Sea and the Japan Island in winter season J Meteor Soc Jpn 1968 46 343 372

[B26] Fukuoka N On the distribution patterns of the so-called Japan Sea elements confined to the Sea of Japan region J Geobotany 1966 15 63 80

[B27] Hotta M History and Geography of Plants. Evolutionary Biology in Plants III 1974 Tokyo: Sanseido

[B28] Shimodaira H An approximately unbiased test of phylogenetic tree selection Syst Biol 2002 51 492 508 10.1080/10635150290069913 12079646

[B29] Mansfield WC Molluscs of the Tampa and Suwannee limestones Geol Bull No 15, State Fla Dep Conserv 1937 15 1 334

[B30] Ladd HS Chitons and Gastropods (Haliotidae through Adeorbidae) from Western Pacific Islands US Geol Surv Professional Pap 1966 531 1 98

[B31] Ponder WF Ponder WF The truncatelloidean (= rissoacean) radiation - a preliminary phylogeny Prosobranc Phylogeny. Malacological Review, Supplement 4 1988 129 166

[B32] Glibert M Revision des Gastropoda du Danien et du Montien de la Belgique. I, Les Gastropoda du Calcaire de Mons Inst R Sci Nat Belg Mem 1973 173 1 115

[B33] Woodruff DS Staub KC Upatham ES Viyanant V Yuan H Genetic variation in *Oncomelania hupensis*: *Schistosoma japonicum *transmitting snails in China and the Philippines are distinct species Malacologia 1988 29 347 361

[B34] Minato H Okutani T Pomatiopsidae Illustrations of Animals and Plants 8. Mollusca 1986 Tokyo Sekaibunka-sya 72 73

[B35] Minato H *Blanfordia integra *Pilsbry, 1924 (Pomatiopsidae); its morphology, ecology, and distribution Nanki Seibutu 1980 22 77 79

[B36] Florens FBV Baider C Relocation of *Omphalotropis plicosa *(Pfeiffer, 1852), a Mauritian endemic landsnail believed extinct J Molluscan Stud 2007 73 205 206 10.1093/mollus/eym004

[B37] Iijima A Tada R Evolution of Tertiary sedimentary basins of Japan in reference to opening of the Japan Sea J Fac Sci, Univ Tokyo, Sec II 1990 22 121 171

[B38] Yonekura N Kaizuka S Nogami M Chinzei K (Eds) Regional Geomorphology of the Japanese Islands. Introduction to Japanese Geomorphology 2001 1 Tokyo: University of Tokyo Press

[B39] Okamoto M Lo CT Tiu WU Qui D Hadidjaja P Upatham S Sugiyama H Taguchi T Hirai H Saitoh Y Habe S Kawanaka M Hirata M Agatsuma T Phylogenetic relationships of snails of the genera *Oncomelania *and *Tricula *inferred from the mitochondrial 12S rRNA gene Jpn J Trop Med Hyg 2003 31 5 10

[B40] Maruyama S Isozaki Y Kimura G Terabayashi M Paleogeographic map of the Japanese Islands: Plate tectonic synthesis from 750Ma to the present The Island Arc 1997 6 121 142 10.1111/j.1440-1738.1997.tb00043.x

[B41] Koike K Tamura T Chinzei K Miyagi T (Eds) Regional geomorphology of the Japanese Islands. Geomorphology of Tohoku Region 2005 3 Tokyo: University of Tokyo Press

[B42] Ekawa K A note on *Blanfordia japonica *from Fukui Prefecture Chiribotan 1985 15 93 97

[B43] Houck MA Bellis ED Comparative tolerance to desiccation in the salamanders *Desmognathus f. fuscus *and *Desmognathus o. ochrophaeus* J Herpetol 1972 6 209 215 10.2307/1562773

[B44] Camp CD Huestis DL Marshall JL Terrestrial versus aquatic phenotypes correlate with hydrological predictability of habitats in a semiterrestrial salamander (Urodela, Plethodontidae) Biol J Linn Soc 2007 91 227 238 10.1111/j.1095-8312.2007.00793.x

[B45] Japan Meteorological Agency Climataic atlas of Japan 2001 Tokyo: Japan Meteorological Agency

[B46] Yamazaki T The plants distribution of Japan Shizenkagaku to Hakubutsukan 1959 26 1 19

[B47] Uemura S Takeda T Nakanishi N Behaviours of the main temperate plants in Hokkaido along climatic gradients Jpn J Ecol 1986 36 141 152

[B48] Shimano K Regeneration dynamics, causal factors, and characteristics of Pacific Ocean-type beech (*Fagus crenata*) forests in Japan: a review Folia Geobot 2002 37 275 296 10.1007/BF02805212

[B49] Bliss LC Adaptations of arctic and alpine plants to environmental conditions Arctic 1962 15 117 144

[B50] Sakai A Hakoda N Cold hardiness of the genus *Camellia* J Am Soc Hortic Sci 1979 104 53 57

[B51] Yoshino MM Natural regions of Japan GeoJournal 1980 4 161 172 10.1007/BF00705523

[B52] Sakai A Larcher W Frost Survival of Plants: Responses and Adaptation to Freezing Stress 1987 Berlin: Springer-Verlag

[B53] Kume A Ino Y Comparison of ecophysiological responses to heavy snow in two varieties of *Aucuba japonica *with different distributing areas Ecol Res 1993 8 111 121 10.1007/BF02348523

[B54] Kume A Tanaka C Adaptation of stomatal response of *Camellia rusticana *to a heavy snowfall environment: Winter drought and net photosynthesis Ecol Res 1996 11 207 216 10.1007/BF02347687

[B55] Suzuki H Uber die Bereiche des winterlichen Niederschlags in Japan Tirigaku Hyouron 1961 34 321 326

[B56] Kaizuka S Late Cenozoic palaeogeography of Japan GeoJournal 1980 4 101 109 10.1007/BF00705517

[B57] Tanai T Graham A Tertiary history of vegetation in Japan Floristics and paleofloristics of Asia and North America 1972 Amsterdam: Elsevier 231 255

[B58] Maekawa F Numata M Origin and characteristics of Japan's flora The Flora and Vegetation of Japan 1974 Tokyo-Amsterdam: Kodansha-Elsevier 33 86

[B59] Kameda Y Kawakita A Kato M Cryptic genetic divergence and associated morphological differentiation in the arboreal land snail *Satsuma *(*Luchuhadra*) *largillierti *(Camaenidae) endemic to the Ryukyu Archipelago, Japan Mol Phylogenet Evol 2007 45 519 533 10.1016/j.ympev.2007.03.021 17500012

[B60] Edgar RC MUSCLE: multiple sequence alignment with high accuracy and high throughput Nucleic Acids Res 2004 32 1792 1797 10.1093/nar/gkh340 15034147 PMC390337

[B61] Castresana J Selection of conserved blocks from multiple alignments for their use in phylogenetic analysis Mol Biol Evol 2000 17 540 552 10742046 10.1093/oxfordjournals.molbev.a026334

[B62] Swofford DL PAUP*. Phylogenetic analysis using parsimony (*and other methods). Version 4.0 2002 Sunderland, MA: Sinauer Associates

[B63] Tanabe AS Kakusan: a computer program to automate the selection of a nucleotide substitution model and the configuration of a mixed model on multilocus data Mol Ecol Notes 2007 7 962 964 10.1111/j.1471-8286.2007.01807.x

[B64] Nixon KC The parsimony ratchet: a new method for rapid parsimony analysis Cladistics 1999 15 407 414 10.1111/j.1096-0031.1999.tb00277.x 34902938

[B65] Vos RA Accelerated likelihood surface exploration: the likelihood ratchet Syst Biol 2003 52 368 373 12775525 10.1080/10635150390196993

[B66] Jobb G TREEFINDERversion of October 2008 2008 Munich, Germany http://www.treefinder.de Distributed by the author at

[B67] Tanabe AS Phylogears version 1.5.2009.12.29, software distributed by the author 2008 http://www.fifthdimension.jp/

[B68] Huelsenbeck JP Ronquist F MRBAYES: Bayesian inference of phylogenetic trees Bioinformatics (Oxf) 2001 17 754 755 10.1093/bioinformatics/17.8.754 11524383

[B69] Ronquist F Huelsenbeck JP MRBAYES 3: Bayesian phylogenetic inference under mixed models Bioinformatics (Oxf) 2003 19 1572 1574 10.1093/bioinformatics/btg180 12912839

[B70] Drummond AJ Ho SYW Phillips MJ Rambaut A Relaxed phylogenetics and dating with confidence PLoS Biol 2006 4 e88 10.1371/journal.pbio.0040088 16683862 PMC1395354

[B71] Drummond AJ Rambaut A BEAST: Bayesian evolutionary analysis by sampling trees BMC Evol Biol 2007 7 214 10.1186/1471-2148-7-214 17996036 PMC2247476

[B72] Minato H Ministry of the Environment Blanfordia integra Threatened Wildlife of Japan: Red Data Book 2nd ed., Land and Freshwater Mollusks 2005 6 Tokyo: Ministry of the Environment 172

[B73] Kawaguchi Y Akita Prefecture Fukuia kurodai kurodai Threatened Wildlife of Akita Prefecture 2002 -Red Data Book of Akita Prefecture 2002 Akita Prefecture 203

[B74] Masuda O Uchiyama R PISCES Ecological Field Gide Series 2: Freshwater Mollusks of Japan 2, Freshwater Mollusks of Japan Including Brackish Water Species 2004 Tokyo: PISCES

[B75] Masuda O Ministry of the Environment Oncomelania nosophora Threatened Wildlife of Japan: Red Data Book 2nd ed., Land and Freshwater Mollusks 2005 6 Tokyo: Ministry of the Environment 68

[B76] Suzuki T Yamashita H Miyagi T Tatara Y *Cecina manchurica *A. Adams, 1861 (Caenogastropoda: Pomatiopsidae) from Miyagi Prefecture, northern Japan Molluscan Divers 2009 1 5 11

[B77] Japan Meteorological Agency Mesh Climatic Data of Japan 2002 Tokyo: Japan Meteorological Business Support Center

