# Supplementary material for: Terrestrial invasion of pomatiopsid gastropods in the heavy-snow region of the Japanese Archipelago
Source: BMC Evol Biol. 2011 May 5;11:118. doi: 10.1186/1471-2148-11-118 (PMC3102040; doi:10.1186/1471-2148-11-118)
Supplement: Additional file 1 — Information on primers and PCR conditions used in this study. [file 1471-2148-11-118-S1.PDF]

Additional File 1 Information on primers and PCR conditions used in this study.

Primer	Direction	Sequence 5'–3'	PCR condition
18S rRNA			
PCR amplification and sequencing			
G01 [1]	Forward	CACCT GGTTG ATCCT GCCAG	94 °C 4 min, (94 °C 30 s, 55 °C 30 s,
G07 [1]	Reverse	AGCTT GATCC TTCTG CAGGT TCACC TAC	72 °C 2 min) x 35, 72 °C 5 min
Sequencing			
G03 [1]	Forward	GTCTG GTGCC AGCAG CCGCG G	
1155F [2]	Forward	CTGAA ACTTA AAGGA ATTGA CGG	
18Sop [3]	Reverse	GCTCC CTCTC CGGAA TCGAA CCC	
G08 [1]	Reverse	GAACG GCCAT GCACC ACCAC C	
28S rRNA			
PCR amplification and sequencing			
28SD1 [4]	Forward	ACCCS CTGAA YTTAA GCAT	94 °C 4 min, (94 °C 30 s, 52 °C 30 s,
28ff [5]	Reverse	GGTGA GTTGT TACAC ACTCC TTAGC GGAT	72 °C 2 min) x 40, 72 °C 5 min
Sequencing			
C2F [6]	Forward	GAAAA GAACT TTGAA GAGAG AGT	
D2F [7]	Forward	CCCGT CTTGA AACAC GGACC AAGG	
C2R [6]	Reverse	ACTCT CTCTT CAAAG TTCTT TTC	
D3 [7]	Reverse	GACGA TCGAT TTGCA CGTCA	
16S rRNA			
16S-H [8]	Forward	CGCCT GTTTA TCAAA AACAT	94 °C 4 min, (94 °C 30 s, 52 °C 30 s,
16S-R [8]	Reverse	CCGGT CTGAA CTCAG ATCAC GT	72 °C 1 min) x 30, 72 °C 5 min
COI			
LCO1490 [9]	Forward	GGT CAA CAA TCA TAA AGA TAT TGG	94 °C 4 min, (94 °C 30 s, 40 °C 30 s,
HCO2198 [9]	Reverse	TAA ACT TCA GGG TGA CCA AAA AAT C	72 °C 2 min) x 30, 72 °C 5 min

References

1. Saunders GW, Kraft GT: **Small-subunit rRNA gene sequences from representatives of selected families of the Gigartinales and Rhodymeniales (Rhodophyta).1. Evidence for the Plocamiales ord. nov.** *Can J Bot* 1994, 72:1250-1263.
2. Wollscheid E, Wa¨gele H: **Initial results on the molecular phylogeny of the Nudibranchia (Gastropoda, Opisthobranchia) based on 18S rDNA data.** *Mol Phylogenet Evol* 1999, 13:215-226.
3. Hoso M, Kameda Y, Wu, SP, Asami T, Kato M, Hori M: **A speciation gene for left-right reversal in snails results in anti-predator adaptation.** *Nat Comm* 2010, 1: 133.
4. Colgan DJ, Ponder WF, Beacham E, Macaranas JM: **Gastropod phylogeny based on six segments from four genes representing coding or non-coding and mitochondrial or nuclear DNA.** *Molluscan Res* 2003, 23: 123-148.
5. Hillis DM, Dixon MT: **Ribosomal DNA: molecular evolution and phylogenetic inference.** *Quart Rev Biol* 1991, 66:411-453.
6. Dayrat B, Tillier A, Lecointre G, Tillier S: **New Clades of Euthyneuran Gastropods (Mollusca) from 28S rRNA Sequences.** *Mol Phylogenet Evol* 2001, 19:225-235.
7. Vonnemann V, Schro¨dl M, Klussmann-Kolb A, Wa¨gele H: **Reconstruction of the Phylogeny of the Opisthobranchia (Mollusca: Gastropoda) by means of 18S and 28S rRNA Gene Sequences.** *J Molluscan Stud* 2005, 71:113-125.
8. Simon S, Frati F, Beckenbach A, Crespi B, Li H, Flook P: **Evolution, weighting and phylogenetic utility of mitochondrial gene sequences and a compilation of conserved polymerase chain reaction primers.** *Ann Ent Soc Am* 1994, 87:651-701.
9. Folmer O, Black M, Hoeh W, Lutz RA, Vrijenhoek R: **DNA primers for amplification of mitochondrial cytochrome c oxidase subunit I from diverse metazoan invertebrates.** *Mol Mar Biol Biotechnol* 1994, 3:294-299.
