# Supplementary material for: Terrestrial invasion of pomatiopsid gastropods in the heavy-snow region of the Japanese Archipelago
Source: BMC Evol Biol. 2011 May 5;11:118. doi: 10.1186/1471-2148-11-118 (PMC3102040; doi:10.1186/1471-2148-11-118)
Supplement: Additional file 2 — Information on sequence alignments and models of sequence evolution for maximum likelihood analysis. [file 1471-2148-11-118-S2.PDF]

Additional File 2 Information on sequence alignments and models of sequence evolution for maximum likelihood analysis.

Alignment	Length of alignment	Excluded sites	Substitution model
18S rDNA	1813	174–196, 244–245, 287, 685, 694–698, 1389, 1393–1395	TN93ef+G
28S rDNA	1536	132–133, 178, 185, 240, 245, 250–251, 448, 480–485, 492–493, 498–499, 505, 520, 535, 539–544, 599–604, 625, 638, 689–693, 722–724, 732, 748–749, 766, 796–797, 801–802, 808–812, 821, 957, 989–1014, 1263, 1366	GTR+G
16S rDNA	550	19–21, 52–54, 59–69, 260–263, 276, 285–288, 307, 344, 356, 378–379, 383, 389, 498, 516	J2+G
COI position 1	223	-	TN93ef+G
COI position 2	223	-	F81+G
COI position 3	223	-	HKY85+G
