# Supplementary material for: Terrestrial invasion of pomatiopsid gastropods in the heavy-snow region of the Japanese Archipelago
Source: BMC Evol Biol. 2011 May 5;11:118. doi: 10.1186/1471-2148-11-118 (PMC3102040; doi:10.1186/1471-2148-11-118)
Supplement: Additional file 3 — Information on localities used for the investigation of climatic factors. [file 1471-2148-11-118-S3.PDF]

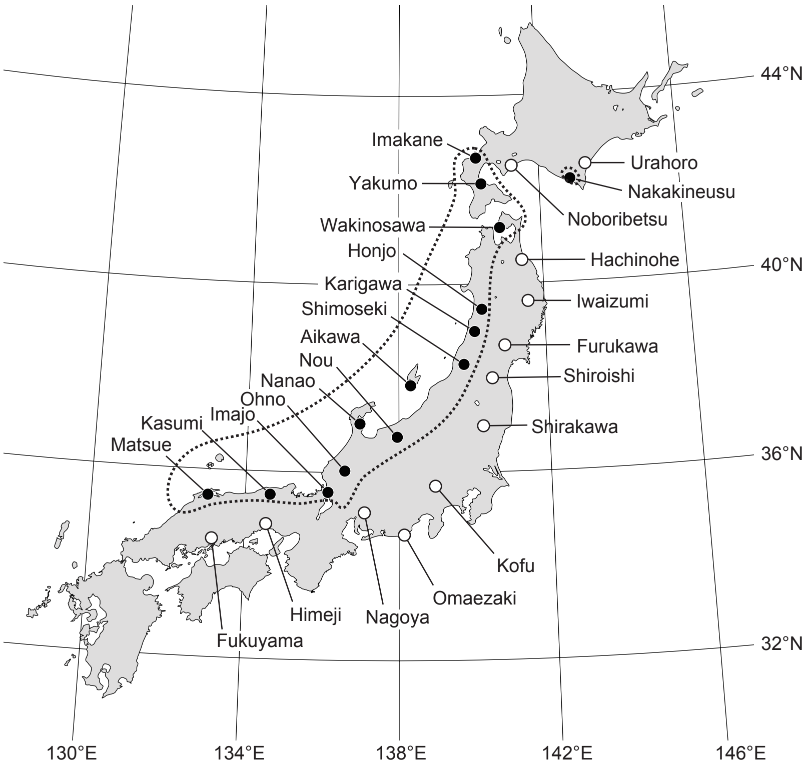

Additional File 3. A map showing localities whose climatic data were used for the logistic regression analysis. Dashed curves represent rough distributions of snails in clades I and II. Solid and open symbols indicate weather stations inside and outside of the distribution.
